# Health Tract, No. 81

**Published:** 1865

**Authors:** 


### HEALTH TRACT. No. 81.

---

### MUSIC

REFINES the taste, purifies the heart, and elevates our nature. It does more: it soothes in sorrow, tranquillizes in passion, and wears away the irritabilities of life. It intensifies love, it fires patriotism, and makes the altar of our devotion burn with a purer, holier flame. Not only man, but the brutes themselves have been restrained and charmed by the bewitching power which it possesses. And in the still twilight hour, when sweet, sad memories go back upon the distant past, and hover lovingly about the places where we played and the persons whom we loved, but now gone, in their youth and beauty and purity, to return no more, who does not know that the soul drinks more deeply in of the saddening sweetness when it breaks out in the soft, low notes of song, or the fingers instinctively sweep through diapasons absolutely ravishing? And when tedious disease has dampened the fires of life, has removed its gilding and written "vanity" on all things earthly; when wealth and fame and worldly honor are felt to be nothing; when the aims and ambitions and aspirations which were wont to rouse up all the energies of nature toward their accomplishment fail of their accustomed power, music renders the burden of sickness light, and makes us all oblivious of pain and suffering. For these reasons, that parent has largely neglected a religious duty, has been strangely forgetful of one of the highest of all obligations, who fails to afford his children, while yet young, all the facilities in his power for fostering and cultivating whatever taste for music they possess, whether vocal or instrumental; for in after-life, and through all its vicissitudes, those who practise it, in the love of it, when young, will find in its exercise a happy escapade in seasons of boisterous mirth, and thus increase the joy; in times of depondency, its expression will give encouragement; when difficulties oppose, it will inspire strength to overcome them, and when clouds of trouble gather around and above, hedging up the future, shutting out the blue sky of life, music can penetrate even Egyptian darkness, and let in upon the almost broken heart the sunshine of hope, of gladness, and of joy.

It is because of this view of the health-giving, happifying, and refining influences of music, that in the progress of a high civilization its cultivation has become a profession, not only among those who give utterance to it in vocal symphonies. "almost divine," but among all classes.

